# 980 Optimizing Burn Patient Care at Discharge: Implementing a New Discharge Process for Improved Outcomes

**DOI:** 10.1093/jbcr/iraf019.511

**Published:** 2025-04-01

**Authors:** Keisha ONeill, Abigail Clark, Mary Kate Messer, ShaNeisha McQueen, Becca Anderson, Kathleen Hollowed, Tiffany Smith, Rohit Mittal, Steven Kahn

**Affiliations:** South Carolina Burn Center at MUSC; South Carolina Burn Center at MUSC; South Carolina Burn Center at MUSC; South Carolina Burn Center at MUSC; South Carolina Burn Center at MUSC; South Carolina Burn Center at MUSC; South Carolina Burn Center at MUSC; Medical University of South Carolina; Medical University of South Carolina

## Abstract

**Introduction:**

Thorough discharge education is vital in optimizing care, particularly for burn patients with complex needs. This study aims to evaluate the quality of a newly developed discharge process in a burn center that treats both adult and pediatric patients. The new process included a more tailored, patient/family centered handouts and education, along with a more detailed discharge check list based on issues identified during Quality Improvement analyses. Analysis focused on satisfaction and patient/family perception of their ability to manage wound care and dressing changes.

**Methods:**

The original discharge process included the traditional written discharge instructions without both handouts and verbal teach back methods. The new process included packets with an educational teach-back guide used to give the patient the opportunity to use a written method to perform teach back and verify products needed for their dressing change at home, as well as visual depictions of specific step by step wound care instructions. Patients were required to demonstrate how to clean the burn wound and change each dressing prior to discharge rather than simply verbalize understanding.

A control group that utilized the old process was surveyed for 30 days prior to implementation of the new process. Patients received a survey at the time of discharge, followed by a repeat survey at their first clinic visit. A 5-point Likert scale survey was used to evaluate patient satisfaction with educational resources, and patient confidence levels after implementation of the new process.

A Mann-Whitney U test was employed to compare survey scores and specifically evaluate the patient confidence level from both phases to assess for statistical significance, using a p-value of < 0.05.

**Results:**

The discharge survey satisfaction scores significantly increased with the new process: 32 control surveys with a mean score of 3.475 and 26 post implementation surveys with a mean score of 4.23 (p=0.0062).

Confidence scores increased from 3.44 to 4.04 (p=0.0477), indicating an improvement in patient/family perceived ability to manage wound care and dressing changes at home.

**Conclusions:**

The new discharge process correlated with higher patient satisfaction scores and increased confidence levels with wound care and dressing changes. Additional study and data collection is needed to determine whether the tools that were implemented improve outcomes and prevent complications post discharge. The surveys also underscore the need for continuous monitoring of patient satisfaction.

**Applicability of Research to Practice:**

Improvement in discharge process to prevent complications after discharge.

**Funding for the Study:**

N/A

